# Genetic Variation in *MDM2* and *p14* ^ARF^ and Susceptibility to Salivary Gland Carcinoma

**DOI:** 10.1371/journal.pone.0049361

**Published:** 2012-11-07

**Authors:** Lei Jin, Li Xu, Xicheng Song, Qingyi Wei, Erich M. Sturgis, Guojun Li

**Affiliations:** 1 Department of Head and Neck Surgery, The University of Texas MD Anderson Cancer Center, Houston, Texas, United States of America; 2 Department of Stomatology, Jinling Hospital, School of Medicine, Southern Medical University, Nanjing, China; 3 Department of Otorhinolaryngology and Head and Neck Surgery, Yuhuangding Hospital of Qingdao University, Yantai, China; 4 Department of Epidemiology, The University of Texas MD Anderson Cancer Center, Houston, Texas, United States of America; IPO, Inst Port Oncology, Portugal

## Abstract

**Background:**

The p14^ARF^/MDM2/p53 pathway plays an important role in modulation of DNA damage and oxidative stress responses. The aim of this study was to determine whether genetic variants in *MDM2* and *p14^ARF^* are associated with risk of salivary gland carcinoma (SGC).

**Methods:**

Four single nucleotide polymorphisms (SNPs) in *MDM2* and *p14^ARF^* (*MDM2*-rs2279744, *MDM2*-rs937283, *p14^ARF^*-rs3731217, and *p14^ARF^*-rs3088440) were genotyped in 156 patients with SGC and 511 cancer-free controls. Multivariate logistic regression analysis was performed to calculate odds ratios (ORs) and 95% confidence intervals (CIs).

**Results:**

*MDM2*-rs2279744 was significantly associated with a moderately increased risk of SGC (OR, 1.5, 95% CI, 1.1–2.2). There was a trend toward significantly increased SGC risk with increasing number of risk genotypes of the four polymorphisms (*P*_trend_ = 0.004). Individuals carrying 3–4 risk genotypes in *MDM2* and *p14^ARF^* were at increased SGC risk (OR, 2.0, 95% CI, 1.1–2.7) compared with individuals carrying 0–2 risk genotypes. Moreover, the combined effect of risk genotypes of *MDM2* and *p14^ARF^* was more pronounced among young subjects (≤45 years), female subjects, subjects with race/ethnicity other than non-Hispanic white, ever-smokers, and ever-drinkers.

**Conclusion:**

Our results support the involvement of SNPs of *MDM2* and *p14^ARF^*, either alone or more likely in combination, in susceptibility to SGC. Larger studies are needed to validate our findings.

## Introduction

Salivary gland carcinoma (SGC) is a relatively rare malignancy with great heterogeneity in histologic features. Apart from exposure to ionizing radiation, which is a confirmed environmental risk factor, the risk factors for SGC are poorly understood [1]. Inherited variations in oncogenes and tumor suppressor genes influence individual susceptibility to a variety of cancers, but their impact on SGC risk has rarely been studied.

MDM2 (murine double minute-2) and p14^ARF^ are the principal cellular regulators of p53 in response to stressors including radiation exposure and exposure to various chemical agents [2]. As a negative regulator of p53, MDM2 not only binds directly to *p53* to block transcriptional activation but also promotes p53 degradation by ubiquitination and proteolysis [3]. In turn, *MDM2* itself is under transcriptional control by p53, such that a negative feedback loop exists between MDM2 and p53. p14^ARF^, a tumor suppressor, participates in the regulatory feedback loop with p53 and MDM2: p14^ARF^ directly interacts with MDM2 to antagonize its inhibition of p53, and p53 in turn represses *p14^ARF^* expression [4], [5]. The autoregulatory feedback loop in the p14^ARF^/MDM2/p53 pathway keeps p53 activity in a delicate balance, which is critical in maintenance of genome integrity and prevention of cancer [3], [6]. Therefore, genetic alterations of *MDM2* and *p14^ARF^* may significantly affect p53-related tumor suppression and subsequently cancer development [7]–[9]. In addition, MDM2 and p14^ARF^ not only interact with p53 but also interact with other important cellular proteins, including Rb, ATM, and E2F/DP1, to exert their oncogenic and tumor suppressor function, respectively [3], [6].

The aim of this case-control study was to determine whether any association exists between polymorphisms in *MDM2* and *p14^ARF^* and risk of SGC. Four functional single nucleotide polymorphisms (SNPs) were selected and genotyped. These particular SNPs were selected because they are common SNPs that have been considered possible susceptibility biomarkers for a variety of cancers [10]–[13]. In the present study, we compared frequency distributions of these four SNPs alone or in combination between cases and controls and evaluated their associations with SGC risk.

## Materials and Methods

### Study Subjects

This case-control study was conducted at The University of Texas MD Anderson Cancer Center as previously described [14]. The study was approved by the Institutional Review Board, and informed consent was obtained from all participants prior to enrollment. The study population included 156 patients with incident SGC (the final diagnosis of SGC was confirmed by histopathology) and 511 cancer-free controls recruited at MD Anderson Cancer Center. The controls were recruited from among visitors to the institution as a control group for a study of molecular epidemiology of squamous cell carcinoma of the head and neck. To be eligible for participation in this study, individuals had to be 18 years or older, without prior malignancy except for nonmelanoma skin cancer, without blood transfusion in the past 6 months, and not taking immunosuppressant medications at the time of recruitment. Each participant donated 20 ml of blood for laboratory analysis and completed a self-administrated questionnaire covering demographic and exposure variables. Race/ethnicity was self-reported and categorized as non-Hispanic white or other; ever-smokers were defined as persons who had smoked more than 100 cigarettes in their lifetime; ever-drinkers were defined as persons who had used alcohol at least once a week for more than 1 year; and radiation exposure was defined as a history of radiotherapy for the treatment of any disease or condition except for current illness.

### SNP Selection and Genotyping Analysis

Four SNPs, *MDM2*-rs2279744, *MDM2*-rs937283, *p14^ARF^*-rs3731217, and *p14^ARF^*-rs3088440, were selected on the basis that they all have a minor allele frequency greater than 10% in Caucasians (dbSNP and SNP500Cancer project, National Cancer Institute); reside in the promoter regions of the genes and therefore have potential functional significance; and have been reported to be associated with cancer risk [10]–[13]. Genomic DNA extracted from the blood samples was used for genotyping analysis and polymerase chain reaction-restriction fragment length polymorphism assay was applied, as described previously [11], [15]. Primers and restriction enzymes used for genotyping analysis are listed in Table S1. Genotyping was performed by laboratory personnel blinded to the case-control status. Results of repeated analysis in a randomly selected subset of 10% of samples were 100% concordant with the results of initial analysis.

### Statistical Analysis

Statistical analyses were performed using SAS software, version 9.2 (SAS Institute Inc., Cary, NC). All statistical tests were two-sided, and *P*<0.05 was accepted as statistically significant. Demographic characteristics were compared between cases and controls using the chi-square test. Hardy-Weinberg equilibrium was evaluated using the chi-square test in the controls for each SNP. Odds ratios (ORs) and 95% confidence intervals (CIs) for risk of SGC were estimated for the studied SNPs, individually and in combination, by multivariate logistic regression with adjustment for age, sex, race/ethnicity, and radiation exposure. The adjustment factors were determined by using stepwise regression (forward selection and backward elimination at the 0.1 alpha level) with age, sex, and race/ethnicity forced to be included. The genotypes whose risk estimate of main effect (e.g., estimated OR from single SNP analysis) was greater than 1 were defined as risk genotypes. Association analyses of the combined genotypes were further stratified by age, sex, race/ethnicity, first-degree family history of cancer, smoking, and alcohol drinking.

## Results

Participant characteristics are summarized in **Table 1**. Cases were more likely than controls to report a history of exposure to medical radiation, but the majority of the subjects did not have such a history. No significant difference was observed between cases and controls in the frequency distributions of sex, race/ethnicity, first-degree family history of cancer, smoking, or alcohol drinking. The most common primary histologic subtypes were adenoid cystic carcinoma (58/156, 37.2%), mucoepidermoid carcinoma (44/156, 28.2%), salivary duct carcinoma/adenocarcinoma not otherwise specified (22/156, 14.1%), and acinic cell carcinoma (11/156, 7.1%).

**Table 1:** Selected demographic and exposure characteristics of cancer-free controls and SGC cases.

	Controls(No. = 511)		Cases(No. = 156)		
Characteristic	No.	(%)	No.	(%)	*P* a
Age, years					<0.001
<48	246	(48.1)	51	(32.7)	
≥48	265	(51.9)	106	(67.3)	
Sex					0.169
Male	245	(47.9)	65	(41.7)	
Female	266	(52.1)	91	(58.3)	
Race/ethnicity					0.943
Non-Hispanic white	401	(78.5)	122	(78.2)	
Other	110	(21.5)	34	(21.8)	
First-degree family history of cancer^b^					0.334
Yes	257	(51.4)	86	(55.8)	
No	243	(48.6)	68	(44.2)	
Smoking^b^					0.307
Ever	213	(42.1)	72	(46.8)	
Never	293	(57.9)	82	(53.2)	
Alcohol drinking^b^					0.764
Ever	246	(48.6)	77	(50.0)	
Never	260	(51.4)	77	(50.0)	
Radiation exposure^b^					0.024
No	504	(98.6)	147	(95.5)	
Yes	7	(1.4)	7	(4.5)	

a Chi-square test, compared to controls.

b Totals are less than the total number of individuals because of missing data.

The distribution of all four SNPs followed Hardy-Weinberg equilibrium (*P*>0.05) in the controls. Genotype frequencies and minor allele frequencies for each SNP in SGC cases and controls are shown in **Table 2**. *MDM2*-rs2279744 G allele was found less frequently among SGC cases than controls (37.5% vs. 44.0%, *P* = 0.041). Comparisons between patients with different histologic subtypes of SGC revealed no significant differences in genotype frequencies of all four SNPs (*P*>0.05) (data not shown).

**Table 2:** *MDM2* and *p14^ARF^* genotype frequencies of cancer-free controls and SGC cases.

	Controls(No. = 511)		Cases(No. = 156)		
Genotype	No.	(%)	No.	(%)	*P* a
*MDM2*-rs2279744					
TT	170	(33.3)	67	(43.0)	0.086
TG	232	(45.4)	61	(39.1)	
GG	109	(21.3)	28	(17.9)	
G allele frequency		(44.0)		(37.5)	0.041
*MDM2-*rs937283					
AA	173	(33.9)	59	(37.8)	0.384
AG	255	(49.9)	68	(43.6)	
GG	83	(16.2)	29	(18.6)	
G allele frequency		(41.2)		(40.4)	0.800
*p14^ARF^*-rs3731217					
TT	388	(75.9)	109	(69.9)	0.204
TG	113	(22.1)	45	(28.8)	
GG	10	(2.0)	2	(1.3)	
G allele frequency		(13.0)		(15.7)	0.225
*p14^ARF^*-rs3088440					
GG	402	(78.7)	125	(80.1)	0.610
GA	98	(19.2)	26	(16.7)	
AA	11	(2.1)	5	(3.2)	
A allele frequency		(11.7)		(11.5)	0.922

a Chi-square test, compared to controls.

The genotype-specific risks for SGC for the four SNPs are presented in **Table 3**. A significant SGC risk was found for *MDM2*-rs2279744 (*P_adj_* = 0.026) but not for the other three SNPs. We further analyzed the genotype-specific risk of each SNP for the primary histologic subtypes and did not find any significant association (data not shown).

**Table 3:** *MDM2* and *p14^ARF^* polymorphisms and risk of SGC.

Genotype	Controls		Cases				*P* a	Adjusted OR^b^ (95% CI)
	(No. = 511)		(No. = 156)					
	No.	(%)		No.		(%)		
*MDM2*-rs2279744								
TG/GG	341	(66.7)	89		(57.0)		0.027	1.0
TT	170	(33.3)	67		(43.0)			1.5 (1.1–2.2)
*MDM2-*rs937283								
AG/GG	338	(66.1)	97		(62.2)		0.363	1.0
AA	173	(33.9)	59		(37.8)			1.2 (0.8–1.8)
*p14^ARF^*-rs3731217								
TT	388	(75.9)	109		(69.9)		0.129	1.0
TG/GG	123	(24.1)	47		(30.1)			1.3 (0.9–2.0)
*p14^ARF^*-rs3088440								
GA/AA	109	(21.3)	31		(19.9)		0.695	1.0
GG	402	(78.7)	125		(80.1)			1.2 (0.7–1.9)

a Chi-square test, compared to controls.

b Adjusted for age, sex, race/ethnicity, and radiation exposure.

To evaluate the combined effect of all four SNPs, participants were further grouped according to the number of risk genotypes. In the present study, the risk genotypes were *MDM2*-rs2279744 TT, *MDM2*-rs937283 AA, *p14^ARF^*-rs3731217 TG/GG, and *p14^ARF^*-rs3088440 GG. As shown in **Table 4**, SGC risk increased with the number of risk genotypes: compared with subjects carrying 0 risk genotypes, subjects carrying one risk genotype had approximately 1.7 times the risk, those carrying two risk genotypes had approximately 2.0 times the risk, those carrying three risk genotypes had approximately 2.7 times the risk, and those carrying four risk genotypes had approximately 5.6 times the risk, indicating a significant trend effect (*P_trend_* = 0.004). When subjects were dichotomized by number of risk genotypes, those with 3–4 risk genotypes (high risk) had approximately twice the risk of those with 0–2 risk genotypes (low risk) (*P_adj_* = 0.022).

**Table 4:** Combined effect of *MDM2* and *p14^ARF^* genotypes on risk of SGC.

No. of combined risk genotypes	Controls		Cases		*P* a	Adjusted OR^b^ (95% CI)
	(No. = 511)		(No. = 156)			
	No.	(%)	No.	(%)		
0	32	(6.3)	5	(3.2)	0.107	1.0
1	181	(35.4)	47	(30.1)		1.7 (0.6–4.8)
2	219	(42.9)	69	(44.2)		2.0 (0.8–5.5)
3	67	(13.1)	27	(17.3)		2.7 (1.0–7.9)
4	12	(2.4)	8	(5.1)		5.6 (1.5–21.0)
						*P_trend_* = 0.004
0–2	432	(84.5)	121	(77.6)	0.043	1.0
3–4	79	(15.5)	35	(22.4)		2.0 (1.1–2.7)

a Chi-square test, compared to controls.

b Adjusted for age, sex, race/ethnicity, and radiation exposure.

To further assess the significance of the association across subgroups, stratification analyses with dichotomized risk genotypes were performed (**Table 5**). Compared with the risk in subjects with 0–2 risk genotypes, the increased risk associated with 3–4 risk genotypes was more pronounced for young subjects (≤45 years) (*P_adj_* = 0.037), female subjects (*P_adj_* = 0.042), subjects with race/ethnicity other than non-Hispanic white (*P_adj_* = 0.007), ever-smokers (*P_adj_* = 0.021), and ever-drinkers (*P_adj_* = 0.011), though no significant interaction term was found among these factors (*P*>0.05).

**Table 5:** Stratification analysis of the combined risk genotypes in association with SGC risk.

Stratification variable	Low-risk genotypes(0–2) (Ref.)		Adjusted OR^a^(95% CI)	High-risk genotypes(3–4)		Adjusted OR^a^ (95% CI)
	Controls	Cases		Controls	Cases	
	No. (%)	No. (%)		No. (%)	No. (%)	
Age, years						
≤45	166 (83.8)	28 (66.7)	1.0	32 (16.2)	14 (33.3)	2.3 (1.1–5.0)
>45	266 (85.0)	93 (81.6)	1.0	47 (15.0)	21 (18.4)	1.4 (0.8–2.6)
Sex						
Female	219 (82.3)	67 (73.6)	1.0	47 (17.7)	24 (26.4)	1.8 (1.0–3.2)
Male	213 (86.9)	54 (83.1)	1.0	32(13.1)	11 (16.9)	1.5 (0.7–3.5)
Race/ethnicity						
Non-Hispanic white	339 (84.5)	99 (81.2)	1.0	62 (15.5)	23 (18.8)	1.3 (0.8–2.3)
Other	93 (84.6)	22 (64.7)	1.0	17 (15.5)	12 (35.3)	3.7 (1.4–8.6)
First-degree familyhistory of cancer						
Yes	319 (85.2)	67 (77.9)	1.0	38 (14.8)	19 (22.1)	1.7 (0.9–3.3)
No	203 (83.5)	52 (76.5)	1.0	40 (16.5)	16 (23.5)	1.5 (0.8–3.0)
Smoking						
Ever	183 (85.9)	54 (75.0)	1.0	30 (14.1)	18 (25.0)	2.3 (1.1–4.7)
Never	245 (83.6)	65 (79.3)	1.0	48 (16.4)	17 (20.7)	1.4 (0.7–2.6)
Alcohol drinking						
Ever	211 (85.8)	57 (74.0)	1.0	35 (14.2)	20 (26.0)	2.4 (1.2–4.6)
Never	217 (83.5)	62 (80.5)	1.0	43 (16.5)	15 (19.5)	1.2 (0.6–2.4)

a Adjusted for age, sex, race/ethnicity, and radiation exposure.

## Discussion

In this study, the combined risk genotypes of *MDM2* and *p14^ARF^* were significantly associated with a moderately increased risk for SGC in a dose-dependent manner. Although our study had limited power to detect modest to moderate increase in risk of cancer by histologic subtype, our results did not show that the observed association was restricted to any specific histologic subtype, which suggests that high-risk genotypes of *MDM2* and *p14^ARF^* might be an indicator of susceptibility to SGC in general.

The association between high-risk genotypes of *MDM2* and *p14^ARF^* and SGC risk was significant among ever-smokers and ever-drinkers but not among never-smokers and never-drinkers. Although no conclusive evidence links smoking and alcohol drinking with risk of SGC, a modest association between smoking and alcohol drinking and increased risk of SGC has been observed [16]. The *p53* gene is the primary target in smoking- and alcohol-associated cancer [17], [18]. Given the role of *MDM2* and *p14^ARF^* in regulating p53 activity, it is reasonable to expect a greater susceptibility to SGC in ever-smokers and ever-drinkers. We also observed a higher frequency of high-risk genotypes of *MDM2* and *p14^ARF^* in young (≤45 years old) patients, suggesting a possible effect of susceptibility to onset age of SGC in this study population. However, these results were obtained from a relatively small number of cases and could be due to chance. High-risk genotypes of *MDM2* and *p14^ARF^* seemed to confer a greater increase in risk for SGC in the race/ethnicity group other than non-Hispanic white, but this group was a small proportion of the study population and consisted of subjects with a variety of ethnic backgrounds. Larger studies from diverse populations are warranted.

We found that *MDM2*-rs2279744 was associated with SGC risk at a nominal significance level of 0.05. *MDM2*-rs2279744, also referred to as *MDM2* SNP309, was originally described as a potential functional SNP biomarker for predisposition to breast cancer [8]. In contrast with previous *in vitro* functional assays and some population-based studies, in which the T allele of rs2279744 was associated with decreased expression levels of MDM2 and decreased risk for cancer [12], [19], the present study showed that the TT genotype of rs2279744 was associated with increased risk of SGC. However, an association between the TT genotype and increased risk of cancer has also been found for risk of prostate cancer and lung cancer [15], [20]. Because cancer is a complex disease caused by a combination of genetic and environmental factors in a progressive manner, risk associated with an SNP may vary greatly with the existence of SNPs in other susceptibility genes and environmental risk factors as well as with types of cancer. In addition, the discrepancies between different studies could be the result of genetic heterogeneity across different populations, a concept that is supported by the observed difference in allele frequency of rs2279744 across different ethnic populations [12].

To the best of our knowledge, this is the first study reporting an association between genetic variants in *MDM2* and *p14^ARF^* and risk of SGC. Several limitations should be noted when interpreting the results: 1) the small sample size increases the possibility that the statistically significant results could be due to chance; 2) the hospital-based case-control study design is susceptible to selection bias; 3) the cases of SGC were histologically heterogeneous and represented more than a single discrete disease; and 4) although the studied SNPs have the potential to affect gene function, they may also be in linkage disequilibrium with other SNPs that could better account for the association. To evaluate how likely the significant results are false-positive, we calculated the false-positive report probability (FPRP) which is estimated by incorporating the prior probability and the estimated magnitude of association (OR, 95% CI) [21]. We considered a prior probability of 0.25 when evidence of both biological plausibility and epidemiological association was fair and a prior probability of 0.01 when both were poor. Given the selection criteria of the investigated SNP in the present study, a prior probability of 0.1 (or more) is considered acceptable. The likelihood that the observed significant association between *MDM2*-rs2279744 and SGC risk is false positive is 18.6%, 40.6% and 88.3% given prior probabilities of 0.25, 0.1 and 0.01, respectively; the statistically significant result is considered as noteworthiness with a FPRP value of <50%. In this study, histological heterogeneity did not likely influence the results of association between the studied SNPs and SGC risk because genotype frequencies of these SNPs were similar between different histological subtypes. Therefore, our findings are preliminary but noteworthy, and larger studies in populations with different race/ethnicity background should be conducted to validate the results, determine whether any additional genetic and environmental factors confound this association, and determine whether associations are restricted to specific histological subtypes.

## Supporting Information

Table S1 Primers and restriction enzymes for genotyping analysis. Primer sequence, restriction enzymes and corresponding restriction fragments length for PCR-restriction fragment length polymorphism assay of the investigated SNPs are provided here.
(DOC)
