# Supplementary material for: Genetic Variation in MDM2 and p14 ARF and Susceptibility to Salivary Gland Carcinoma
Source: PLoS One. 2012 Nov 7;7(11):e49361. doi: 10.1371/journal.pone.0049361 (PMC3492289; doi:10.1371/journal.pone.0049361)
Supplement: Table S1 — Primers and restriction enzymes for genotyping analysis. Primer sequence, restriction enzymes and corresponding restriction fragments length for PCR-restriction fragment length polymorphism assay of the investigated SNPs are provided here. (DOC) [file pone.0049361.s001.doc]

Table S1. Primers and restriction enzymes for genotyping analysis

| **SNPs** | **Primer Sequences (5’🡪3’)** | **Restriction Enzyme** | **Restriction Fragment Length (bp)** |
| --- | --- | --- | --- |
| *MDM2*-rs2279744 | S: GTTTTGTTGGACTGGGGCTA  AS: TGCGATCATCCGGACCTCCCGCGTC | TaqI | G: 175  T: 26, 149 |
| *MDM2*-rs937283 | S: TGACCGAGATCCTGCTGCTTTC  AS: TGAGTCAACCTGCCCACTGAAC | BclI | G: 115  A: 17, 98 |
| *p14^ARF^*-rs3731217 | S: AAAAGGGGGACAACCATTCTC  AS: CCCCTCTCAAATATGCTGTCC | MvaI | T: 280  G: 126, 154 |
| *p14^ARF^*-rs3088440 | S: TGCTCACTCCAGAAAACTCCA  AS: ATGTGCCACACATCTTTGACC | HaeIII | A: 356  G: 141, 215 |
